# Study on Demulsification via Vacuum Filtration with Superamphiphilic Diatomite/G-C_3_N_4_/Rice Husk Charcoal Composite Filter Layer

**DOI:** 10.3390/nano15050344

**Published:** 2025-02-22

**Authors:** Yue Wang, Tianxin Chen, Yu Jia, Feng Qin, Junhui Gao, Xingyang Zhang, Jiahong He, Jian He

**Affiliations:** 1 Research Institute of Safety, Environmental Protection and Technical Supervision, PetroChina Southwest Oil & Gasfield Company, Chengdu 610041, China; qhsewyue@petrochina.com.cn (Y.W.); chentianxin@petrochina.com.cn (T.C.); 2 The Quality, Safety, and Environmental Protection Department, PetroChina Southwest Oil & Gas Field Company, Chengdu 610056, China; jiayu10@petrochina.com.cn; 3 Tight Oil and Gas Exploration and Development Project Department, PetroChina Southwest Oil & Gas Field Company, Chengdu 610051, China; qinfeng01@petrochina.com.cn; 4 Northwest Sichuan Gas District, PetroChina Southwest Oil & Gas Field Company, Jiangyou 621700, China; gaojunhui@petrochina.com.cn; 5 School of Chemical Engineering, Sichuan University, Chengdu 610065, China; 2023323070030@stu.scu.edu.cn (X.Z.); 2024323070027@stu.scu.edu.cn (J.H.)

**Keywords:** superhydrophilic, superoleophilic, tight gas, flowback fluid, demulsification

## Abstract

The primary extraction way for unconventional oil/gas resources is hydraulic fracturing to alter the reservoir for commercial production. However, hydraulic fracturing technology consumes a large amount of water, and the flowback water can easily be mixed with hydrocarbon substances to form emulsions. To achieve the recycling of water, it is necessary to develop an efficient continuous demulsification method for treating the flowback fluid. In this study, a composite filtration layer with superhydrophilic and superoleophilic properties was successfully prepared using water-based polyurethane as a binder. The g-C_3_N_4_ was used to improve the affinity of the filtration layer to water and oil. The diatomite and rice husk carbon were used as an adsorbent and a filter aid, respectively. The contact angles (CA) of both oil and water on the surface of the filtration layer were measured to be 0°. During the demulsification process, vacuum filtration was employed to increase the pressure difference across the filtration layer, thereby improving the treatment flux of flowback fluid. The experimental results showed that the filtration flux with the addition of rice husk charcoal increased from 160.58 L∙m^−2^∙h^−1^ to 174.68 L∙m^−2^∙h^−1^ compared to the filter layer without rice husk charcoal. Based on the composite filtration layer, the apparent demulsification efficiency exceeded 90.6% for various types of emulsion. The mechanism of demulsification was investigated by the molecular dynamics method. The results showed that the adsorption layer density of water molecules reached 1.5 g/cm^3^, and the adsorption layer density of oil molecules exceeded 2.5 g/cm^3^. The porous structure wall has a strong adsorption effect on both oil and water molecules, resulting in deformation and destruction of the oil–water interface, so that the dispersed phase is adsorbed and aggregated by the filter layer at the same time and permeates from the filter layer after reaching saturation, thus separating the two phases.

## 1. Introduction

The advancement of oil and gas exploit technology, coupled with the depletion of conventional oil reserves, has led to an increase in unconventional oil and gas resources [1,2]. The proportion of world unconventional oil and gas production in total production may increase from 10% to 30% in 2040. However, the unconventional oil and gas resources are primarily stored in tight rock crevices, and the resources are dispersed. These reservoirs need to be transformed using reservoir modification techniques to achieve commercial production, with hydraulic fracturing being the primary method currently employed [3,4]. The fracturing fluid produced by a single well is approximately 10,000~25,000 m^3^, and approximately 60–80% of the flowback fluid returns to the ground after fracturing [5,6]. To reduce the wastage of water and environmental contamination, it is imperative to treat the fracturing flowback fluid. However, the fracturing flowback fluid is a complex multiphase system comprising guanidine gum, salts, petroleum, and other additives [7,8,9]. The additives are primarily thickeners, surfactants, and drag reducers [10,11], and the fracturing flowback fluid can form emulsions easily, resulting in a complex composition. The discharge of wastewater without prior treatment can not only cause environmental contamination but also cause the waste of valuable resources. The flowback fluid is characterized by a high degree of emulsification, which presents a significant challenge in the recovery of oil resources. The properties of emulsification vary considerably, making it essential to adjust treatment processes accordingly.

The existing fracturing fluid demulsification methods mainly include physical methods [12,13], biological methods [14,15], and chemical demulsification methods [16]. The latest research findings indicate that the emulsified oil/water mixtures can be processed continuously using super-wetting materials, particularly for surfactant-stabilized oil/water emulsions. The demulsification methods utilizing super-wetting materials encompass both filtration and adsorption. In these methods, the pore structure and wettability of the super-wetting materials are of critical importance to the efficiency of demulsification and the flux of water-in-oil emulsions [17]. The formation of micropores in these porous materials typically occurs through the following three different methods. The first involves directly utilizing the material with micropores as the substrate material and modifying the wettability [17,18,19,20]. The second method involves compressing three-dimensional porous super-wetting materials with loose pore sizes, such as super-wetting sponges [21,22,23] and cotton [24,25,26,27]. The third method is the direct synthesis of a new type of super-wetting membrane with micro-pores using chemicals [28].

However, when porous materials are used for demulsification, the pores of the porous materials are prone to blockage by emulsion droplets, resulting in a decline in separation efficiency. Particularly when this phenomenon persists, it may lead to the forced shutdown of the demulsification process. A feasible demulsification method is to apply additional pressure to reduce pore blockage. The utilization of a superhydrophilic diatomite/graphite carbon nitride (g-C_3_N_4_) composite layer, as proposed by He et al. [29], has the potential to facilitate the rapid breaking of water-in-oil emulsions. The use of waterborne polyurethane (PU) glue facilitates the connection of micron-sized porous diatomite into a 3D superhydrophilic/superoleophilic PU-diatomite. The demulsification efficiency of water-in-oil emulsions can reach 97.36% under a vacuum of 20 kPa. In industrial filtration operations, the addition of a large pore filter is a common method to improve filtration efficiency. Rice husk carbon, a carbon material derived from biomass with a porous structure and high porosity, has been extensively employed as a filter aid component. Tuaprakone et al. [30] prepared and obtained a rice husk carbon filter aid with a surface area of 174.95 m^2^/g, which has a good adsorption effect and filtration aid effect. It is therefore possible to ensure the demulsification efficiency of the filtration layer and improve the demulsification rate of the flowback fluid. Therefore, the demulsification process of oil-in-water wastewater can be enhanced by adding rice husk carbon as a porous skeleton filler, which has the potential for industrial application.

In this study, 3D porous superhydrophilic and superoleophilic filter cakes were prepared by using water-based adhesive and hydrophilic particles as demulsification materials. The demulsification efficiency of the filter layer was improved by vacuum suction and adding rice husk carbon as a filter aid. The effect of filter layer composition on the demulsification efficiency of oil-in-water emulsion was investigated. The effects of g-C_3_N_4_ and diatomite on the demulsification process were investigated by molecular simulation. At the same time, the porous rice husk carbon was used as the skeleton support structure to investigate the effect of rice husk carbon additions on the demulsification process of the superhydrophilic filter layer. The factors that may affect the demulsification effect were investigated, including vacuum degree, emulsion particle size, oil–water ratio, and emulsifier content.

## 2. Materials and Method

### 2.1. Materials

BASF waterborne polyurethane dispersion BY-20 was obtained from Guangdong Hongke Chemical Raw Materials Co., Ltd. (Dongguan, China). Hexadecyl dimethyl hydroxypropyl sulfobetaine (40%) was obtained from Shandong Yousuo Chemical Technology Co., Ltd. (Heze, China). 3-sulfopropyl tetradecyl dimethyl betaine (98%) was purchased from Ron Reagent Flagship Store. Coconut oil amide propyl betaine was purchased from Henan Yingfu Chemical (Anyang, China). Rice husk was purchased from Yongsheng Liang Rice Processing Co., Ltd. (Chenzhou, China). Melamine was purchased from Sichuan Meifeng Chemical Co., Ltd. (Deyang, China). Potassium chloride and diatomite were purchased from Chengdu Kelong Chemical Co., Ltd. (Chengdu, China). Flowback fluid was collected from an oil and gas well in Sichuan Province.

### 2.2. Preparation of Superamphiphilic Filter Layer

The wettability of the composite filter layer has a significant effect on the efficiency and flux of the demulsification process, which is controlled by the composition of the filter layer. The g-C_3_N_4_ is used as a filter modifier and has a significant effect on the wettability of the filter layer. The g-C_3_N_4_ is prepared by the following steps: potassium chloride (KCl, 5.96 g) and melamine (3.00 g) were uniformly mixed in a crucible and placed in a muffle furnace. The mixture was heated from room temperature at 5 °C/min to 550 °C for 4 h. After the heating process, the sample was cooled with the furnace. The g-C_3_N_4_ was washed five times with boiling water and dried at 80 °C for 12 h to obtain g-C_3_N_4_. The rice husk carbon was prepared by the following steps. The rice husk (3.00 g) was heated in the crucible from 50 °C to 300 °C at a rate of 5 °C/min in a muffle furnace, and the rice husk carbon was obtained after holding for 2 h and cooling with the furnace. During the heating process, the N_2_ was used as the protective gas to prevent the material from being oxidized.

Finally, the composite filter layer was prepared with the following steps. The g-C_3_N_4_ (0.40 g) and waterborne polyurethane dispersion (4.00 g) were dispersed in deionized water (22.00 g). After that, the rice husk carbon (2.00 g) and diatomite (20.00 g) were added and stirred. The mixture was evenly spread in a Buchner funnel and dried in an oven at 80 °C for 24 h to obtain a filter layer. The thickness of the composite filter layer is around 1 ± 0.2 cm.

### 2.3. Parameters of the Emulsification Process

**Figure 1:**
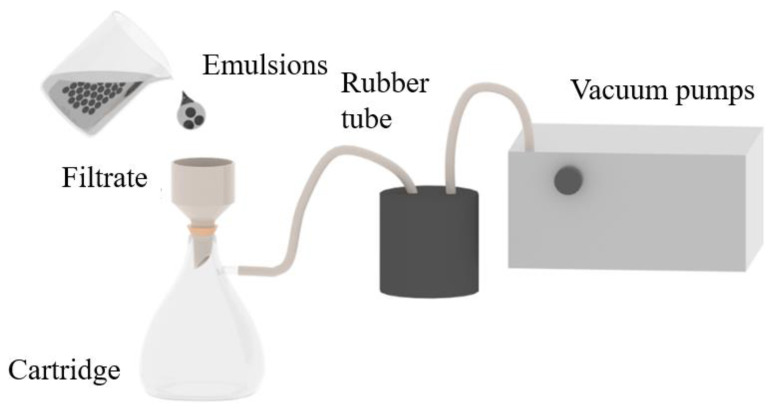
Schematic diagram of the demulsification process based on the composite filter layer and vacuum filtration.

The oil and deionized water (total mass: 100.00 g) collected from the backflow fluid in a tight gas production were mixed with an emulsifier with a mass fraction of 0.1 wt.%. The homogenizer was used to prepare the emulsion at a speed of 7000 rpm for 3 min. The demulsification process was performed with the equipment constructed with the composite filter layer and vacuum filtration shown in Figure 1. The rubber tubes and vacuum pump were used to increase the pressure difference of the filter layer during the filtration process, thereby improving filtration efficiency. The transmittance of the sample in the wavelength range of 600~1000 nm was measured using an ultraviolet–visible spectrophotometer (Lambda750S, PerkinElmer, Waltham, MA, USA). In this process, the transmittance of the deionized water was used as the background. The droplet state was observed by an optical microscope (59XC, Shanghai No. 1 Optical Instrument Factory, Shanghai, China), and the droplet size was measured and counted using ImageJ-IJ1.45 software.

The apparent demulsification efficiency (*η*) is defined as the average transmittance in the wavelength range of 600~1000 nm. The equation for calculating the demulsification efficiency (*ղ_e_*) according to the light transmittance is as follows:

(1) $$\eta_{e}=\frac{m_{1e}}{{m_{0}}_{e}}$$

where *ղ_e_* is the demulsification efficiency, %; *m*_0_*_e_* is the average transmittance of continuous-phase water as a medium before emulsification, *m*_1_*_e_* is the average transmittance of the continuous phase collected after demulsification.

The average demulsification speed *u_e_* per unit time is calculated as follows equation:

(2) $$u_{e}=\frac{V_{e}}{t_{e}}$$

where *u_e_* is the average demulsification rate per unit time, mL/min; *V_e_* is the volume of filtrate collected per unit time, mL; *t_e_* = 5 min, timed by stopwatch.

### 2.4. Molecular Simulation

**Figure 2:**
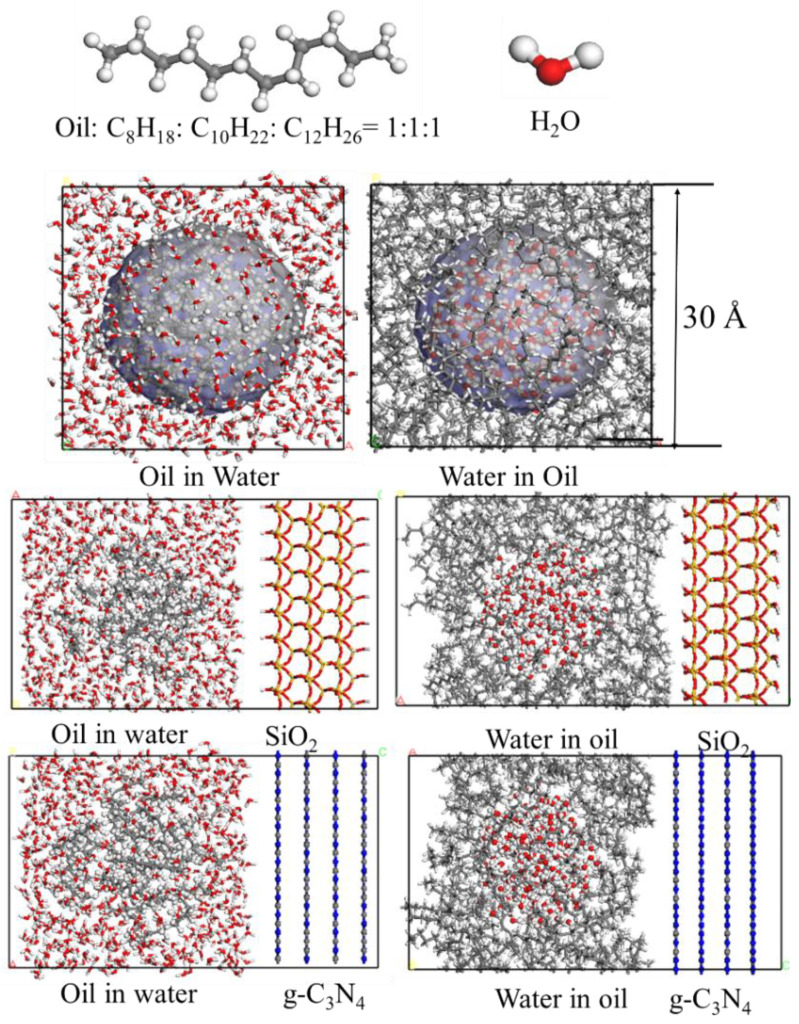
Construction of water-in-oil and oil-in-water emulsion models, and initial molecular structure models of water-in-oil and oil-in-water emulsions distributed in pores constructed by SiO_2_ and g-C_3_N_4_.

To explore the demulsification process of the superamphiphilic filter layer, the molecular dynamics (MD) with the Forcite module in Materials Studio 7.0 (MS-7.0) software was used to investigate the demulsification process of the oil-in-water emulsion. The alkanes of C_8_H_18_, C_10_H_22_, and C_12_H_26_ were used to represent the oil in the fracturing flowback fluid, and the alkanes were added to the pores according to the molar ratio of 1:1:1. The density of oil and water is set as 0.8 g/cm^3^ and 1.0 g/cm^3^. The temperature of the system is set as 298.15 K. The molecular structure of the emulsion is shown in Figure 2. The (001) crystal face of SiO_2_ and g-C_3_N_4_ were used as the pore wall surface, respectively. The interaction between oil and water molecules on the wall was investigated at the molecular scale to clarify the demulsification mechanism. The initial model of the emulsion in the pores is shown in Figure 2.

The initial radius of the oil droplet is 10 Å, and the water molecules are distributed around the oil droplet to construct an initial model of oil in water with dimensions of 30 × 30 × 30 Å. The defect of the 001 crystal plane of SiO_2_ is compensated by the hydroxyl groups. The initial structure model is constructed by combining the droplet model and the cleaved surface, as illustrated in Figure 2. The slit pore size is predominantly maintained within the range of 30 Å to 40 Å. The observed fluctuations in the pore structure are primarily attributed to the extended nature of the oil molecular chain, with some space reserved during the modeling process. Subsequently, the constructed molecular structure is subjected to geometry optimization. Following the optimization process, a canonical ensemble (NVT) MD simulation is conducted with periodic boundary conditions employed throughout the calculation. The total calculation time is 1 ns; the 500 ps in the first stage is used to equilibrate the structure, and the last 500 ps is used to collect the data. The force field of COMPASS is used to control the interaction between the molecules. The charge of the atoms is also distributed according to the force field parameters. Then, the dynamic behavior of oil droplets in water and the competitive adsorption of water and oil on the surface were investigated. After performing molecular dynamics simulation, the density distribution of different components within the pore structure is extracted to examine the adsorption capacity of the porous structure.

## 3. Results and Discussion

### 3.1. Construction and Characterization of Superamphiphilic Filtration Layer

**Figure 3:**
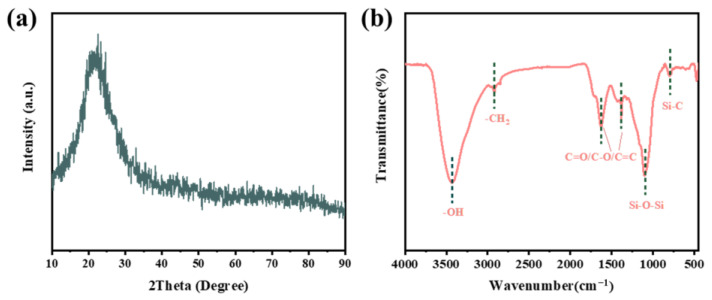
Rice husk carbon prepared at 300 °C (**a**) XRD plot, (**b**) FT-IR analysis.

The crystal structure of rice husk carbon prepared at 300 °C is shown in Figure 3a. X-ray diffraction (XRD) analysis indicates that rice husk carbon exhibits a broad absorption peak at 22°, indicating an amorphous carbon structure. The chemical groups of rice husk carbon were identified through Fourier transform infrared spectroscopy (FT-IR) analysis, as illustrated in Figure 3b. These include the -OH group at 3435 cm^−1^, the -CH_2_ group at 2919 cm^−1^, the C=O and C-O groups at 1628 cm^−1^ and 1384 cm^−1^, and several non-reactive inert functional groups, such as Si-O-Si and Si-C, which were observed at 1104 cm^−1^ and 803 cm^−1^, respectively. The aforementioned analysis results demonstrated that the rice husk is transformed into amorphous carbon and combined with silica following calcination.

**Figure 4:**
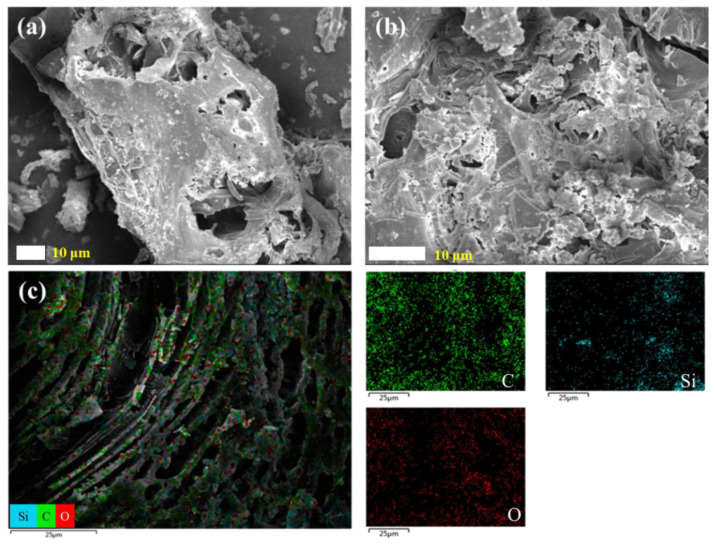
SEM images of RHC: (**a**) 1000×, (**b**) 2000×, (**c**) EDS images.

The morphology of rice husk carbon after pyrolysis is shown in Figure 4a,b. When pyrolyzed at 300 °C, the rice husk carbon still maintained the columnar structure of the fresh rice husk, but the surface was converted to a porous structure. It was also found that the internal structure of rice husk charcoal was mainly distributed with micron pores, containing a variety of staggered distribution channels, which was conducive to the penetration of fluid. The EDS element analysis of the surface is shown in Figure 4c. The result shows that the rice husk carbon mainly contains C, Si, and O, in which the C and O are evenly distributed, while Si elements were aggregated locally.

Based on the previous demulsification filter layer [29], three different types of filter layers were constructed. The main difference between the three types of filter layers is the composite. The first one is constructed with a diatomite and waterborne polyurethane filter layer. The diatomite was used as an adsorbent. The filter exhibits superhydrophilic and oleophilic properties. The second sample is also a composite filter layer constructed with diatomite, g-C_3_N_4,_ and waterborne polyurethane. The addition of g-C_3_N_4_ can improve the wettability of the filter to water and oil. The last sample is constructed with diatomite, rice husk carbon, g-C_3_N_4,_ and waterborne polyurethane. The rice husk carbon was used as a filter aid.

**Figure 5:**
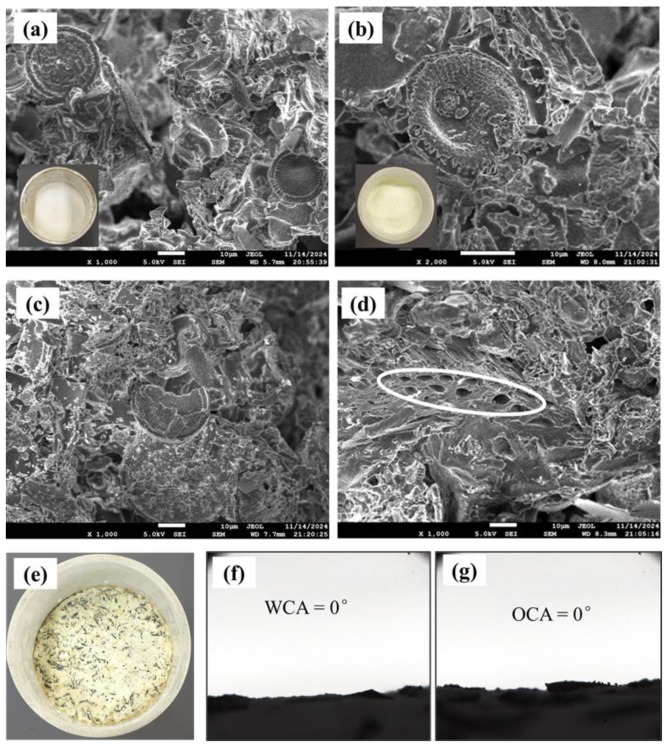
Morphology of composite filter layers observed by SEM: (**a**) diatomite filter layer, (**b**) diatomite and g-C_3_N_4_ composite filter layer; composite filter layer after incorporating rice husk carbon: (**c**) microstructure of the surface, (**d**) interfacial morphology, (**e**) macroscopic morphology of surface; contact angle of the composite filter layer after incorporating rice husk carbon: (**f**) water, (**g**) oil.

To investigate the changes in the structure of the filter layer before and after the addition of rice husk carbon, the microstructure and surface element composition of the prepared composite filter layer were analyzed. The microstructure of the composite filter layers with different compositions is shown in Figure 5a–d. Based on the bonding effect of waterborne polyurethane glue, the single filter layer structure with diatomite as filler is shown in Figure 5a. Diatomite particles with different geometric structures are accumulated to form a porous structure, especially the circular diatoms with a diameter of about 30 μm that maintain the original structure of diatoms. After doping with g-C_3_N_4_, the morphology of the filter layer changed little (Figure 5b). The color changed from gray to light yellow, which was mainly caused by the uniform distribution of light-yellow g-C_3_N_4_. After the addition of rice husk, the morphology of composite filter layers is shown in Figure 5c. Diatomite and rice husk carbon accumulate with each other to form a new composite filter layer. A large number of micron-sized pore structures can be observed in the cross-section of the filter layer (Figure 5d). Black rice husk carbon was slowly added during the preparation of the filter layer, thus evenly distributed throughout the filter layer. Therefore, the addition of the rice husk carbon introduces more micron-sized porous structures to the composite filter layer, providing abundant channels for oil and water to detach from the filter layer after demulsification, thereby increasing the filtration rate. The destruction of the oil–water interface of the droplets and the adsorption of emulsifiers provide abundant sites.

The wettability of the filter layer represents a crucial determinant in the process of demulsification. The contact angles of the composite filter layer doped with rice husk carbon were evaluated using water (WCA) and oil (OCA), respectively. The WCA and OCA were measured using a contact angle meter (JC2000C1, Powereach, Shanghai, China). The results are presented in Figure 5f,g. The results demonstrate that oil and water droplets exhibit rapid spreading behavior when they contact the surface. The contact angles of the water and oil are both 0°, and the interface of the droplets is not discernible on the surface. The results demonstrate that the filter layer prepared by this process exhibits excellent superhydrophilic and superoleophilic properties, which facilitate the wetting ability and adsorption of the continuous phase by the filter layer during the demulsification process. In turn, this disrupts the stability of the oil–water interface, thereby achieving rapid demulsification of the emulsion.

**Figure 6:**
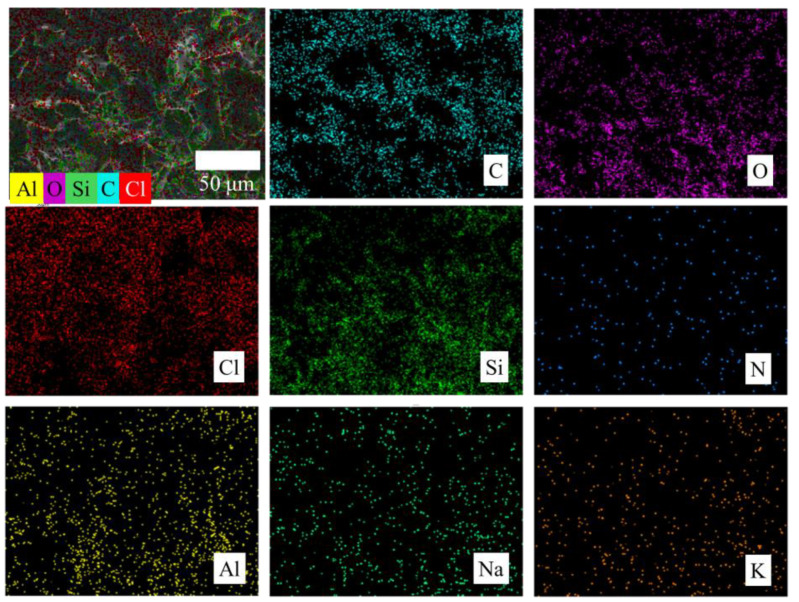
The EDS mapping of the composite filtration layer.

At the same time, the surface of the composite filter layer was subjected to elemental analysis to determine the distribution of different components in the filter layer. The results are shown in Figure 6. From the diagram, it can be seen that there are many kinds of elements on the surface of the filter layer, which are evenly distributed. Among them, Si, Al, O, Na, and other elements are from diatomite. The elements of K, Cl, C, and N are from g-C_3_N_4_ modified by potassium chloride. Another part of C, N, and O comes from waterborne polyurethane resin, and rice husk charcoal also has a large amount of C and a small amount of Si/O elements. Therefore, the composition and structure of the composite filter layer are complex, but it can be found from elemental analysis that different components are evenly distributed in the filter layer, which is crucial to the performance stability of the demulsification process.

**Figure 7:**
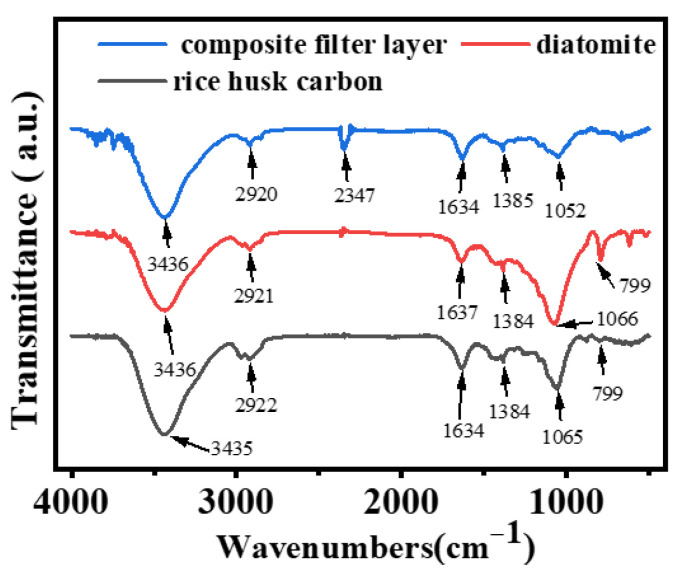
The FT-IR analysis of the composite layer.

To further confirm the chemical composition and pore structure distribution of the filter layer, the analysis of the filter layer with the FT-IR analyzer is shown in Figure 7. The absorption peaks of diatomite and rice husk carbon are basically the same. This is because rice husk carbon also contains silicon and organic matter. The difference is that the absorption peak of the C–H bond at 1634 cm^−1^ is stronger than that in rice husk carbon. The composite filter layer has a stretching vibration peak of the N–H bond in g-C_3_N_4_ at 3436 cm^−1^ [31]. A stretching vibration peak of -CH_3_ in the quaternary ammonium group of waterborne polyurethane at 2920 cm^−1^, an absorption peak of CO_2_ in the air at 2347 cm^−1^. A characteristic peak of C–H in waterborne polyurethane can be detected at 1634 cm^−1^. The characteristic peak of the azine ring in g-C_3_N_4_ is located at 1385 cm^−1^, and a vibration absorption peak of the Si–O–Si at 1052 cm^−1^ should belong to rice husk carbon [32].

### 3.2. Demulsification Efficiency of Superamphiphilic Filter Layer

**Figure 8:**
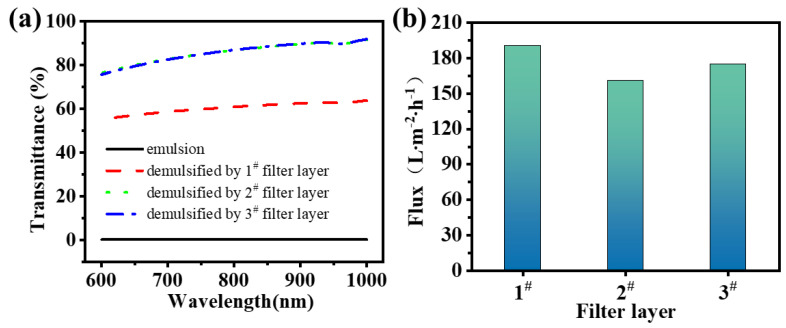
Demulsification of different filter layers: (**a**) transmittance after demulsification, (**b**) flux of different filter layers.

A vacuum filtration demulsification device based on a superamphiphilic filter layer was constructed in accordance with the specifications outlined in Figure 8, with the vacuum maintained at 8 kPa throughout the filtration process. A 50 mL emulsion was prepared for the demulsification process, and the results are presented in Figure 8. The result showed that the demulsification process with diatomite (1#) demonstrated a demulsification flux of 190.48 L·m⁻^2^·h⁻^1^. However, the transmittance test indicated that the demulsification efficiency was only 60.41% after two cycles of demulsification. Following the addition of g-C_3_N_4_ (2#), the emulsification flux was reduced to 160.58 L·m⁻^2^·h⁻^1^, but the emulsification efficiency was markedly enhanced to 85.83%. This illustrates that the amphiphilic property of g-C_3_N_4_ can markedly enhance the demulsification efficiency. Finally, the addition of rice husk carbon (3#) resulted in a negligible change in the demulsification efficiency of the composite filter layer, while the flux was enhanced to 174.68 L·m⁻^2^·h⁻^1^ and 85.78%, representing a marked improvement over the filter layer without rice husk carbon.

### 3.3. Effect of Emulsifier on the Demulsification Process

**Figure 9:**
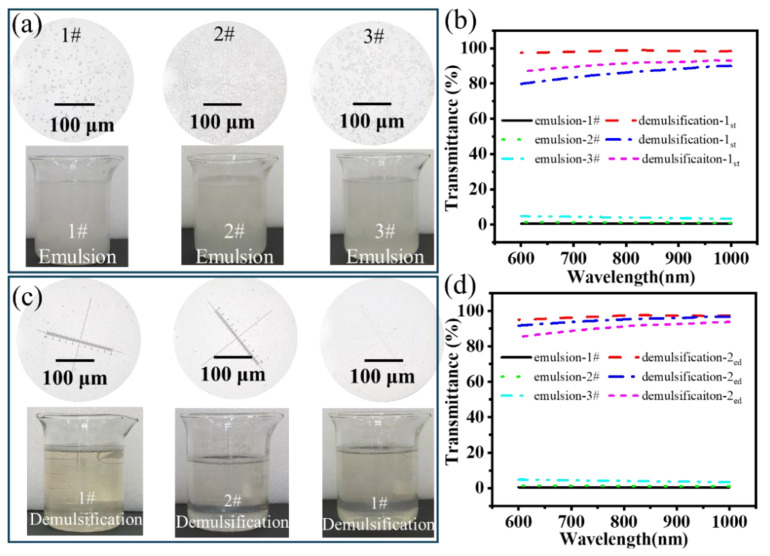
The emulsion with different types of surfactants: (**a**) the appearance of emulsion, (**b**) comparison of transmittance between emulsion and after the first demulsification, (**c**) appearance and microscopic observation results after the second demulsification, (**d**) comparison of transmittance between emulsion and after the second demulsification.

Due to the complex composition of the fracturing flowback fluid, some surfactants may affect the demulsification process. This section explores the effect of emulsifiers on the demulsification efficiency. The betaine series emulsifiers were selected as the research object, which is commonly used in oilfield fracturing fluids. The cetyl/octadecyl dimethyl hydroxypropyl sulfobetaine (1#), coconut oil amide propyl betaine (2#), and 3-sulfopropyl tetradecyl dimethyl betaine (3#) were added to the solution, and the addition amount was 0.1 wt.%. After mixing the three betaine surfactants with the oil–water mixture using a homogenizer, a uniform emulsion was formed, appearing turbid and milky white, as shown in Figure 9a. A large number of micron-sized emulsion droplets can be observed by microscope (59XC, Shanghai No. 1 Optical Instrument Factory). The UV–visible spectrophotometer (LAMBDA 750S, PerkinElmer, USA) was used to detect the transmittance of the emulsion. As shown in Figure 9b, the light transmittance in the wavelength range of 600–1000 nm is less than 3%. After one-time demulsification by the rice husk carbon/diatomite composite filter, the light transmittance exceeded 80%. After two times of suction filtration demulsification shown in Figure 9c,d, the filtrate gradually became clear, and the presence of emulsion droplets was almost not observed under a microscope. After two times of demulsification, the apparent demulsification rates of the filter layer on the three emulsions reached 96.8%, 94.8%, and 90.6%, respectively. Therefore, the type of emulsifier has a certain influence on the demulsification efficiency of the filter layer, and the filter layer containing rice husk has the best demulsification effect to the emulsion mixed with 1# emulsifier.

**Figure 10:**
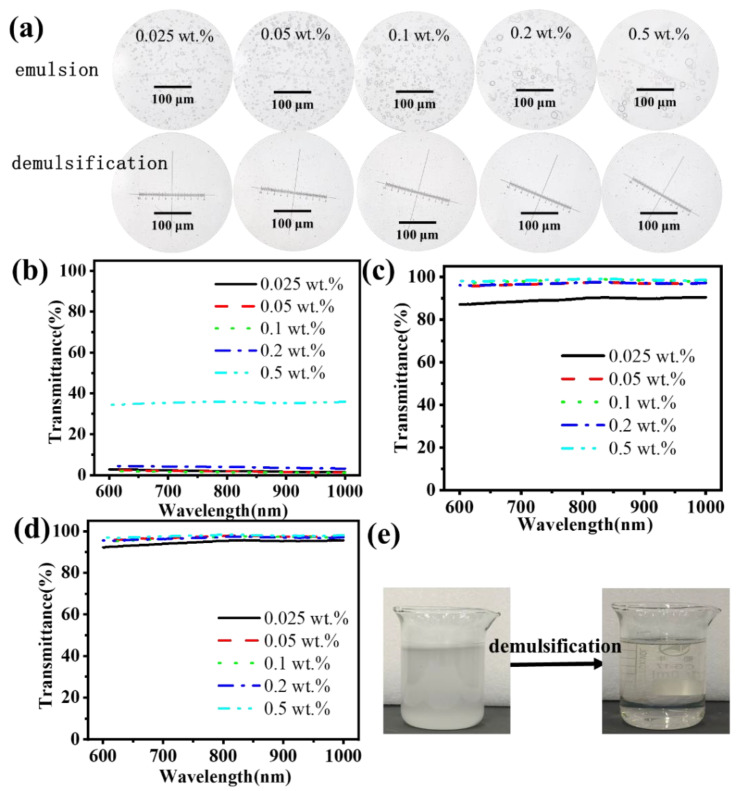
The emulsion with different qualities of surfactants: (**a**) emulsion state under a microscope before and after demulsification; light transmittance analysis of wastewater after demulsification: (**b**) initial emulsion, (**c**) first time, (**d**) second time. (**e**) Appearance of emulsion containing 0.5 wt% surfactant and after demulsification.

To further investigate the impact of emulsifiers on the system, five different mass fractions of emulsifier were selected for examination. As illustrated in Figure 10a,b, an increase in emulsifier content is accompanied by a gradual increase in droplet size. In turn, a notable increase in turbidity and a corresponding decrease in transmittance can be observed. However, when the content of the emulsifier was increased to 0.5 wt.%, the transmittance of the emulsion increased to 35%. This was due to the excessive addition of the emulsifier, which resulted in a portion of the emulsifier being dispersed unevenly. In turn, this also accelerated the settling process of the droplets, and a decrease in the number of droplets was also observed through the microscope.

After two cycles, the transmittance is illustrated in Figure 10c. After the first filtration process, the amount of emulsifier greater than 0.05 wt%, the emulsion after the demulsification exhibited a transmittance of over 90%. When the emulsifier content is reduced, the droplet particle size of the emulsion is also reduced. The transmittance was observed to be only 89%. Following two cycles of demulsification, the emulsion breakage rate of the various emulsifier emulsions exceeded 94.7% (Figure 10d). The filtrate exhibited minimal emulsion droplets (Figure 10a), becoming a clarified and transparent wastewater (Figure 10e). The experimental results demonstrated that the super-wetting composite filtration layer exhibited an excellent demulsification effect for wastewater with varying degrees of emulsification.

### 3.4. Effect of the Particle Size Distribution of Emulsion

**Figure 11:**
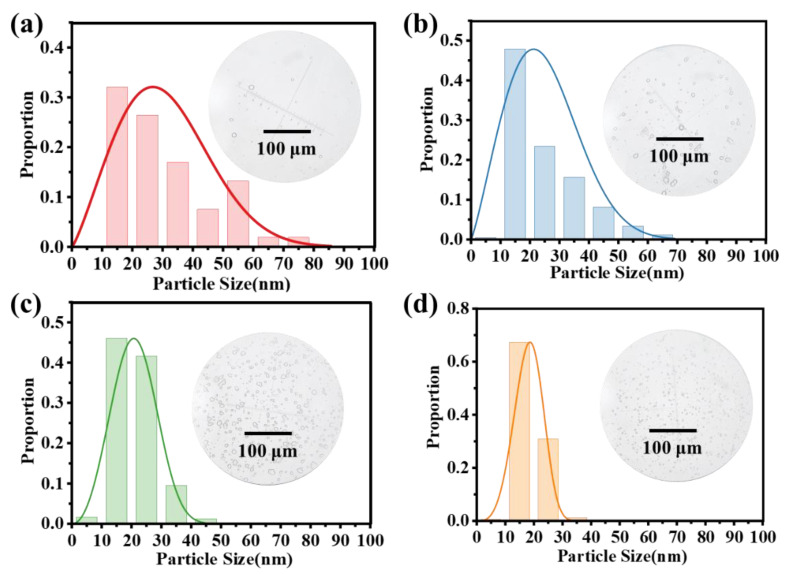
Particle size distribution of emulsions prepared at different homogenization speeds: (**a**) 3 krpm, (**b**) 5 krpm, (**c**) 7 krpm, (**d**) 9 krpm.

By modifying the rotational velocity of the high-speed homogenizer, the particle size distribution of the demulsification can be modified to investigate the impact of droplet size on the filtration breaking process. By adjusting the rotational speed, emulsions with different particle size distributions can be obtained, as illustrated in Figure 11a–d. The emulsions are white and translucent, and the number of droplets is increased with the increase in the rotational speed under microscopic observation. Additionally, the number of large-size droplets in emulsions decreases gradually. Furthermore, increasing the speed of the homogenizer led to a small range of particle size distribution in the emulsions. The range decreased from 10–80 μm at 3 krpm to 10–30 μm at 9 krpm. The average particle size decreased from 30.88 μm to 18.16 μm.

**Figure 12:**
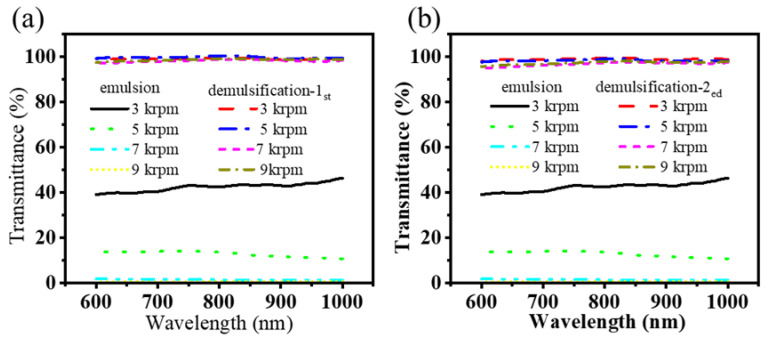
Light transmittance analysis of emulsions with different particle sizes after demulsification: (**a**) initial emulsion and demulsification for the first time; (**b**) initial emulsion and demulsification for the second time.

Nevertheless, as the stirring speed of the homogenizer increases, the particle size of the emulsion gradually decreases, accompanied by a reduction in transparency from approximately 40% to approximately 1–3%, as illustrated in Figure 12a. With a single demulsification process, it can be observed that the composite filter layer exhibits a notable demulsification effect on the emulsion produced at varying speeds, with a demulsification efficiency exceeding 98.2%. After two cycles of demulsification, the demulsification efficiency exceeded 96.8%, as illustrated in Figure 12b. The treated wastewater was clear and transparent, and no emulsion droplets were observed under the microscope. Even the transmittance of some emulsions was observed to be lower than that of the emulsion for the first demulsification. This phenomenon may be attributed to the removal of surfactants from the wastewater and oil mixture during the demulsification process, which subsequently passes through the filter layer. The large pore size of filter layers may facilitate the formation of emulsions, leading to the generation of new emulsions and a reduction in the transmittance of the emulsion.

### 3.5. Effect of Oil Content and Vacuum Degree

**Figure 13:**
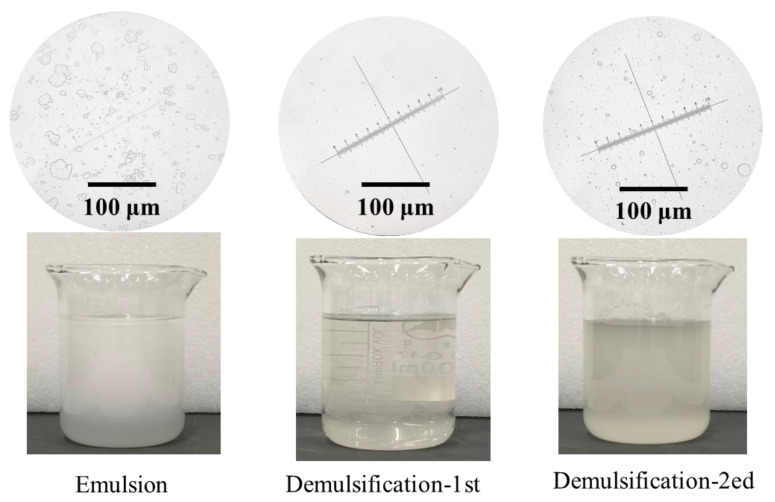
The appearance of emulsions with different oil contents in wastewater and the corresponding state after demulsification.

The oil content in different flowback fluids varies greatly. To investigate the demulsification effect of the composite filter layer on oil-in-water emulsions with different oil contents, five emulsions with different oil contents were selected for vacuum filtration demulsification experiments. The amount of oil added to the water was 0.1 wt.% to 5 wt.%. The appearance of the emulsion with 5 wt.% oil after rapid shearing by a homogenizer is shown in Figure 13. After high-speed shearing, the emulsion was transformed into an opaque, milky white state. Compared to the emulsion with a mass fraction of 0.1 wt.%, it can be found by the microscope that the number of dispersed droplets in the emulsion decreased, but the size of the oil droplets increased. This may be due to the further increase in the number of dispersed phases, resulting in the coalescence of dispersed phases and a subsequent increase in the size of the emulsion droplets. After the first demulsification, the emulsion became clear, and the droplets were scarcely observed in the microscope. However, after the second demulsification, the emulsion became turbid again, and the presence of droplets can be clearly observed under the microscope.

**Figure 14:**
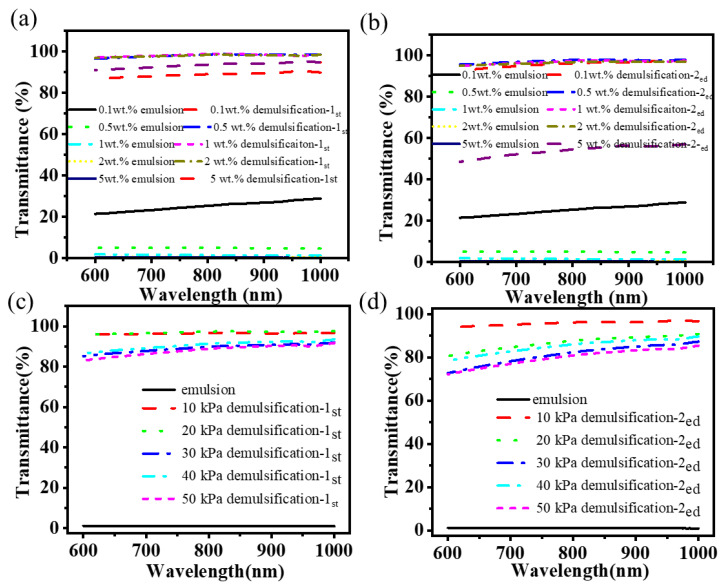
Transmittance analysis of emulsions with different oil after demulsification: (**a**) initial emulsion, (**b**) first time, (**c**) second time; transmittance analysis of emulsions demulsified at different vacuum levels: (**c**) first time; (**d**) second time.

The results of the transmittance of the emulsion are illustrated in Figure 14a. As the oil content of the emulsion increases, the transmittance of the emulsion also increases, reaching a maximum at a certain point and then decreasing. After a single demulsification process, the light transmittance of all emulsions exhibited a notable increase. However, for emulsions with an oil content exceeding 2 wt.%, the light transmittance remained below 90%. The results of the secondary demulsification process are presented in Figure 14b. Except for the emulsion with an oil content of 5 wt.%, the demulsification rate of the composite filter layer on the other emulsions with oil content is above 95.7% after two cycles of demulsification. In the case of the emulsion with an oil content of 5 wt.%, the emulsion droplets were also observed in the water after two cycles of demulsification. This phenomenon may be attributed to the saturation of the adsorption of the oil phase by the composite filter layer. During the demulsification process, the water mixed with the oil again, thereby reforming the emulsion droplets. It can therefore be concluded that the 3D superhydrophilic and superoleophilic filter layer not only exhibits demulsification capabilities but also demonstrates an emulsification effect when the adsorption of the filter layer reaches saturation. It is of great importance to optimize the parameters of the demulsification process to achieve efficient demulsification.

The vacuum degree has an impact on the residence time of the emulsion within the composite filter layer. It can be observed that an increase in vacuum degree results in a corresponding increase in driving force, which in turn leads to an acceleration in the flow rate of the emulsion within the filter layer and a reduction in the residence time. As illustrated in Figure 14c, the initial emulsion exhibits minimal transmittance, whereas the composite filter layer subjected to demulsification with varying vacuum degrees displays a notable enhancement in transparency. Nevertheless, as the vacuum degree increased gradually, the demulsification effect diminished. The results of the transmittance analysis are presented in Figure 14c,d.

Following a single demulsification process, the demulsification efficiency exceeded 88.1%. Following a second demulsification process, the minimum demulsification efficiency was reduced to 80.0%. This may be attributed to an increase in the vacuum degree. The residence time is reduced, which hinders the full contact between the micro-emulsion and the filter layer, thereby impeding the demulsification process. As the emulsion passes through the filter layer, the continuous phase is continually extracted, increasing the number of emulsions and decreasing light transmittance. Therefore, it can be observed that there is a contradiction between the vacuum degree and the demulsification effect. It is essential to select an appropriate vacuum degree in accordance with the actual processing flow.

### 3.6. Analysis of Demulsification Mechanism

**Figure 15:**
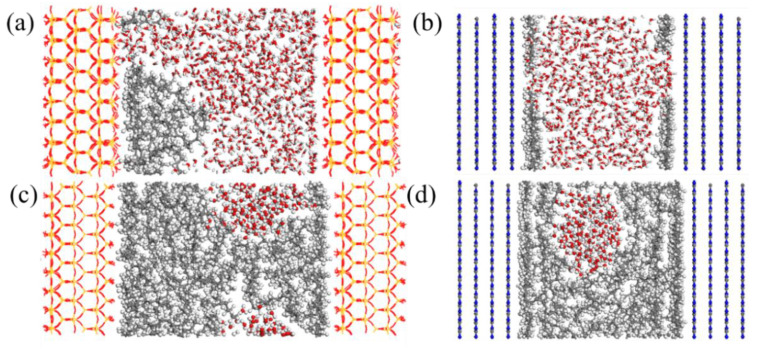
Equilibrium states of water-in-oil emulsions in different types of pores: (**a**) SiO_2_, (**b**) g-C_3_N_4_; equilibrium states of oil-in-water emulsions in different types of pores: (**c**) SiO_2_, (**d**) g-C_3_N_4_.

The composite filter layer, comprising g-C_3_N_4_, rice husk carbon, and diatomite, exhibits a demulsification performance for oil-in-water emulsions. This is primarily attributable to the superoleophilic and superhydrophilic characteristics of the composite filter layer. As shown in Figure 15a–d, the SiO_2_ was used to represent the diatomite in the MD simulations. The result demonstrated that when the SiO_2_ is initially wetted by water, it hinders the spreading of oil droplets on the surface. The oil droplet on the surface exhibits a hemispherical state, which is readily displaced by water from the surface. However, the surface of g-C_3_N_4_ displays a stronger affinity for oil in both oil-in-water and water-in-oil emulsions. In the presence of a relatively low oil content, water can contact the surface. As the oil content increases, it becomes increasingly challenging for water to interact with the surface, resulting in significant adsorption of oil onto the pore surface.

**Figure 16:**
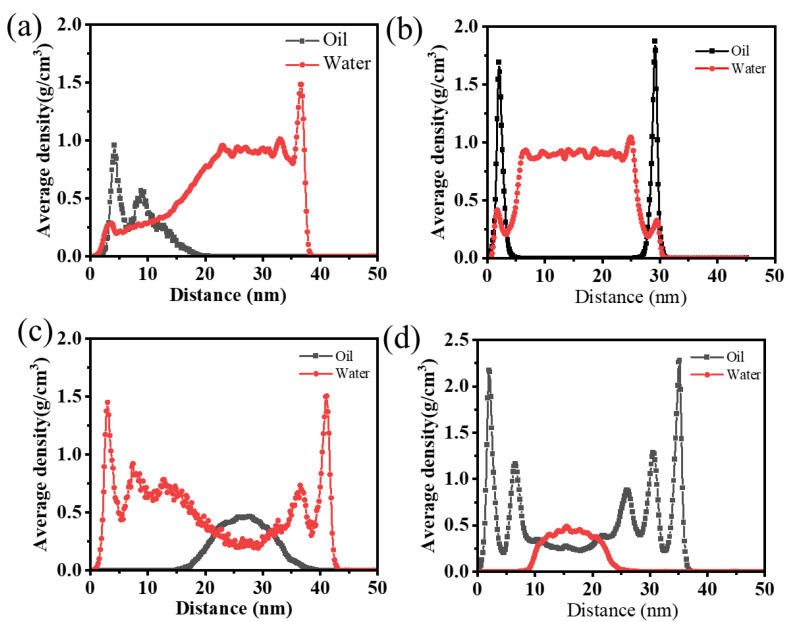
Density distribution of water-in-oil emulsions in different pore channels: (**a**) SiO_2_; (**b**) g-C_3_N_4_; Density distribution of oil-in-water emulsions in different pore channels: (**c**) SiO_2_; (**d**) g-C_3_N_4_.

To enhance comprehension of the adsorption of oil and water by the two materials, the average density of water and oil in the cross-section along the vertical axis of the channel was calculated. The results are presented in Figure 16. As can be observed in Figure 16a,c, the adsorption layer density of water on the surface of SiO_2_ reaches 1.5 g/cm^3^. In the case of an oil-in-water emulsion, the oil droplets remain aggregated on the surface of SiO_2_, indicating that they have not yet fully dispersed. Consequently, the number of oil molecules present is relatively limited. Additionally, water molecules are distributed on the surface of SiO_2_ and penetrate the interstitial space between the oil and the surface. The calculated average density of water molecules is 0.2 g/cm^3^.

The adsorption of oil molecules on the surface of g-C_3_N_4_ differs significantly from that on the surface of SiO_2_. As illustrated in Figure 16b,d, for the oil-in-water emulsion, the density of the adsorption layer reaches 1.75 g/cm^3^, and the distribution density of oil-free molecules in the middle of the pores is zero, while the density of water molecules in the middle of the pores is 0.9–1 g/cm^3^. Two distinct adsorption layers are observed on the surface of the water-in-oil emulsion g-C_3_N_4_. The density of the initial adsorption layer reaches 2.25 g/cm^3^, while the density of the subsequent layer reaches 1.3 g/cm^3^. These findings illustrate the remarkable adsorption capacity of oil molecules by g-C_3_N_4_. In contrast, the average density of water molecules is approximately 0.5 g/cm^3^ only in the central region of the pores. Therefore, it can be concluded that the composite filter layer prepared by diatomite and g-C_3_N_4_ exhibits excellent superamphiphilic and adsorption properties, which enable the separation and aggregation of oil after demulsification.

**Figure 17:**
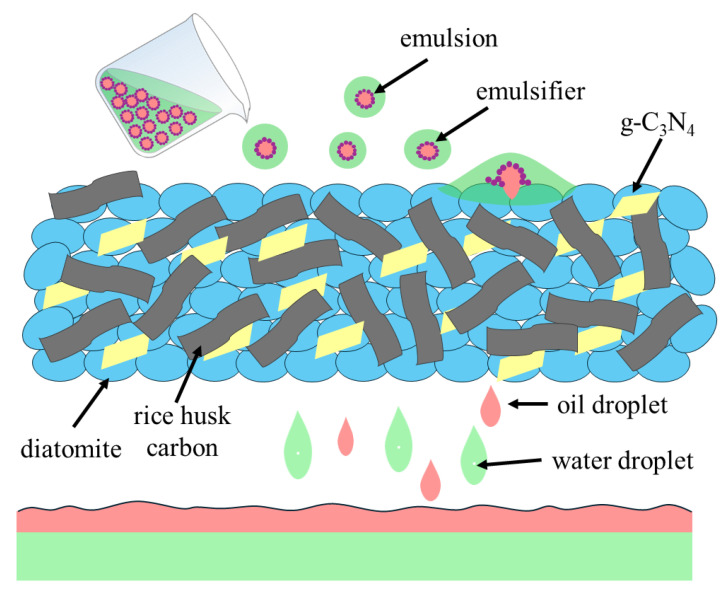
The demulsification mechanism of 3D composite filter layer.

The mechanism of demulsification is illustrated in Figure 17. The emulsion present in the flowback fluid is an oil-in-water emulsion, and the composite filter layer has a rich micro-nano pore structure, which enables rapid and continuous demulsification. Upon contact with the filter layer, the oil-in-water emulsion is subjected to deformation and destruction owing to the adsorption of the surface. The water in the emulsion rapidly spreads on the surface of the filter layer to form a water film, and the internal oil droplets are also adsorbed by the filler. Once the water phase has reached saturation by the hydrophilic filter layer, water is continuously exuded from the filter layer through the pores by means of gravity and vacuum suction. At the same time, the oil droplets reach saturation point and gradually penetrate the filter layer in the form of a continuous phase. The filter layer’s porous structure and high specific surface area provide a conducive environment for the coalescence of oil and water. As the surfactant, which is responsible for maintaining the stability of the oil–water interface, passes through the complex micron-sized pores, it is adsorbed or confined to the porous structure. The stability of the oil–water interface in the emulsion droplets is compromised, resulting in the aggregation of oil droplets from multiple particles to form a two-phase system. This process also facilitates the demulsification process of suction filtration.

## 4. Conclusions

In this study, a composite filter layer was developed based on diatomite/g-C_3_N_4_/rice husk charcoal, which can be used for the demulsification of oil–water emulsions in fracturing flowback fluid. The oil–water contact angle on the composite filter layer is zero, showing a superamphiphilic property. The impact of surfactants, varying addition levels, and the demulsification process on demulsification efficacy was evaluated. The result showed that the addition of rice husk carbon enhanced the demulsification efficiency of the filter layer. The filtration flux of rice husk carbon exhibited an increase from 160.58 L·m⁻^2^·h⁻^1^ to 174.68 L·m⁻^2^·h⁻^1^. The oil-in-water emulsions stabilized by different emulsifiers demonstrated a notable demulsification effect, with apparent demulsification rates higher than 90.6%. For the emulsion with a different particle size oil, the demulsification rate of the filter layer following a single demulsification process exceeds 98.2%. Nevertheless, the demulsification capacity of the filter layer is markedly influenced by the oil concentration and residence time. The demulsification rate of the emulsion with a low oil content is only above 95.7% after two times of demulsifications, whereas the demulsification rate decreases by 80% when the oil content in the wastewater exceeds 5 wt.%. The results of the MD simulations demonstrate that the diatomite and g-C_3_N_4_ exhibit a pronounced adsorption effect on water and oil. The adsorption layer density of water is 1.5 g/cm^3^, while that of oil is 2.5 g/cm^3^. Therefore, the demulsification process within the filter layer is primarily dependent on the adsorption capabilities of the filter layer, which disrupts the stability of the oil–water interface.

## Data Availability

Data are contained within the article.

## References

[1] Muther T., Qureshi H.A., Syed F.I., Aziz H., Siyal A., Dahaghi A.K., Negahban S. (2022). Unconventional hydrocarbon resources: Geological statistics, petrophysical characterization, and field development strategies. J. Pet. Explor. Prod. Technol..

[2] Aadnøy B.S., Looyeh R., Aadnøy B.S., Looyeh R. (2019). Chapter 16—Shale Oil, Shale Gas, and Hydraulic Fracturing. Petroleum Rock Mechanics.

[3] Zhang X.-S., Wang H.-J., Ma F., Sun X.-C., Zhang Y., Song Z.-H. (2016). Classification and characteristics of tight oil plays. Pet. Sci..

[4] Li Z., Wang J., Gates I.D. (2020). Fracturing Gels as Analogs to Understand Fracture Behavior in Shale Gas Reservoirs. Rock Mech. Rock Eng..

[5] Stewart C.B., Lowes H.M., Mehler W.T., Snihur K.N., Flynn S.L., Alessi D.S., Blewett T.A. (2023). Spatial and temporal variation in toxicity and inorganic composition of hydraulic fracturing flowback and produced water. J. Hazard. Mater..

[6] Rivard C., Lavoie D., Lefebvre R., Séjourné S., Lamontagne C., Duchesne M. (2014). An overview of Canadian shale gas production and environmental concerns. Int. J. Coal Geol..

[7] Willems D.J., Kumar A., Nugegoda D. (2022). The Acute Toxicity of Salinity in Onshore Unconventional Gas Waters to Freshwater Invertebrates in Receiving Environments: A Systematic Review. Environ. Toxicol. Chem..

[8] Golding L.A., Kumar A., Adams M.S., Binet M.T., Gregg A., King J., McKnight K.S., Nidumolu B., Spadaro D.A., Kirby J.K. (2022). The influence of salinity on the chronic toxicity of shale gas flowback wastewater to freshwater organisms. J. Hazard. Mater..

[9] Chang H., Li T., Liu B., Vidic R.D., Elimelech M., Crittenden J.C. (2019). Potential and implemented membrane-based technologies for the treatment and reuse of flowback and produced water from shale gas and oil plays: A review. Desalination.

[10] Stringfellow W.T., Domen J.K., Camarillo M.K., Sandelin W.L., Borglin S. (2014). Physical, chemical, and biological characteristics of compounds used in hydraulic fracturing. J. Hazard. Mater..

[11] Vidic R.D., Brantley S.L., Vandenbossche J.M., Yoxtheimer D., Abad J.D. (2013). Impact of Shale Gas Development on Regional Water Quality. Science.

[12] Chang H., Liu S., Tong T., He Q., Crittenden J.C., Vidic R.D., Liu B. (2020). On-Site Treatment of Shale Gas Flowback and Produced Water in Sichuan Basin by Fertilizer Drawn Forward Osmosis for Irrigation. Environ. Sci. Technol..

[13] Miller D.J., Huang X., Li H., Kasemset S., Lee A., Agnihotri D., Hayes T., Paul D.R., Freeman B.D. (2013). Fouling-resistant membranes for the treatment of flowback water from hydraulic shale fracturing: A pilot study. J. Membr. Sci..

[14] Akyon B., Stachler E., Wei N., Bibby K. (2015). Microbial Mats as a Biological Treatment Approach for Saline Wastewaters: The Case of Produced Water from Hydraulic Fracturing. Environ. Sci. Technol..

[15] Butkovskyi A., Bruning H., Kools S.A.E., Rijnaarts H.H.M., Van Wezel A.P. (2017). Organic Pollutants in Shale Gas Flowback and Produced Waters: Identification, Potential Ecological Impact, and Implications for Treatment Strategies. Environ. Sci. Technol..

[16] da Silva Duarte J.L., Solano A.M.S., Arguelho M.L., Tonholo J., Martínez-Huitle C.A., e Silva C.L.D.P. (2018). Evaluation of treatment of effluents contaminated with rifampicin by Fenton, electrochemical and associated processes. J. Water Process Eng..

[17] Yue X., Fu D., Zhang T., Yang D., Qiu F. (2020). Superhydrophobic Stainless-Steel Mesh with Excellent Electrothermal Properties for Efficient Separation of Highly Viscous Water-in-Crude Oil Emulsions. Ind. Eng. Chem. Res..

[18] Liu W., Cui M., Shen Y., Zhu G., Luo L., Li M., Li J. (2019). Waste cigarette filter as nanofibrous membranes for on-demand immiscible oil/water mixtures and emulsions separation. J. Colloid Interface Sci..

[19] Brown P.S., Bhushan B. (2015). Bioinspired, roughness-induced, water and oil super-philic and super-phobic coatings prepared by adaptable layer-by-layer technique. Sci. Rep..

[20] Gao S.J., Shi Z., Zhang W.B., Zhang F., Jin J. (2014). Photoinduced Superwetting Single-Walled Carbon Nanotube/TiO_2_ Ultrathin Network Films for Ultrafast Separation of Oil-in-Water Emulsions. ACS Nano.

[21] Wang C.-F., Chen L.-T. (2017). Preparation of Superwetting Porous Materials for Ultrafast Separation of Water-in-Oil Emulsions. Langmuir ACS J. Surf. Colloids.

[22] Zhang J., Zhao J., Qu W., Li X., Wang Z. (2020). One-step, low-cost, mussel-inspired green method to prepare superhydrophobic nanostructured surfaces having durability, efficiency, and wide applicability. J. Colloid Interface Sci..

[23] Yu T., Halouane F., Mathias D., Barras A., Wang Z., Lv A., Lu S., Xu W., Meziane D., Tiercelin N. (2020). Preparation of magnetic, superhydrophobic/superoleophilic polyurethane sponge: Separation of oil/water mixture and demulsification. Chem. Eng. J..

[24] Li F., Bhushan B., Pan Y., Zhao X. (2019). Bioinspired superoleophobic/superhydrophilic functionalized cotton for efficient separation of immiscible oil-water mixtures and oil-water emulsions. J. Colloid Interface Sci..

[25] Li T., Liu F., Zhang S., Lin H., Wang J., Tang C.Y. (2018). Janus Polyvinylidene Fluoride Membrane with Extremely Opposite Wetting Surfaces via One Single-Step Unidirectional Segregation Strategy. ACS Appl. Mater. Interfaces.

[26] Chauhan P., Kumar A., Bhushan B. (2019). Self-cleaning, stain-resistant and anti-bacterial superhydrophobic cotton fabric prepared by simple immersion technique. J. Colloid Interface Sci..

[27] Wang Z., Wang Y., Liu G. (2016). Rapid and Efficient Separation of Oil from Oil-in-Water Emulsions Using a Janus Cotton Fabric. Angew. Chem. Int. Ed..

[28] Yang X., Ma J., Ling J., Li N., Wang D., Yue F., Xu S. (2018). Cellulose acetate-based SiO_2_/TiO_2_ hybrid microsphere composite aerogel films for water-in-oil emulsion separation. Appl. Surf. Sci..

[29] He Y., Zhang X., Wei J., Wang W., Li Z., Wang P., Jia X., Zhao P. (2024). Rapid demulsification of water in oil emulsion of supernitriphilic carbon-diatomite composite filter layer. Chem. Eng. Oil Gas.

[30] Tuaprakone T., Wongphaet N., Wasanapiarnpong T. (2011). Fabrication of activated rice husk charcoal by slip casting as a hybrid material for water filter aid. IOP Conf. Ser. Mater. Sci. Eng..

[31] Zhai H., Jia L., Yang W., Wu P., He J., Liu C., Jiang W. (2023). Effect of *g*-C_3_N_4_ morphology on its performance as lubricating additive for grease. Colloids Surf. Physicochem. Eng. Asp..

[32] Caliskan N., Kul A.R., Alkan S., Sogut E.G., Alacabey İ. (2011). Adsorption of Zinc(II) on diatomite and manganese-oxide-modified diatomite: A kinetic and equilibrium study. J. Hazard. Mater..

